# The changes in various hydroxyproline fractions in aortic tissue of rabbits are closely related to the progression of atherosclerosis

**DOI:** 10.1186/1476-511X-9-26

**Published:** 2010-03-09

**Authors:** Mohamed Anwar K Abdelhalim, NJ Siddiqi, AS Alhomida, Mohammed S Al-Ayed

**Affiliations:** 1 Department of Physics and Astronomy, College of Science, King Saud University, P.O. Box 2455, Riyadh-11451, Saudi Arabia; 2 Department of Biochemistry, College of Science, King Saud University, P.O. Box 2455, Riyadh-11451, Saudi Arabia

## Abstract

**Background:**

The most important function of collagen and elastin is to induce several mechanical parameters which are known to play a dominant role in governing mechanical properties of the blood vessels. The aortic tissue of rabbit is one of the important sources of collagen and elastin. The effects of high fat diet (HFD) on the hydroxyproline (Hyp) fractions in serum and aortic tissues of rabbits and collagen content in the aortic tissues of rabbits have not been documented before. The present study was undertaken to investigate the changes in Hyp fractions in serum and aortic tissues of rabbits and collagen content in the aortic tissues of rabbits during the progression of atherosclerosis. The atherosclerotic model used in this study was the New Zealand white rabbit (male; 12 weeks old). Twenty five rabbits were individually caged, and divided into control group (NOR; n = 10) and HFD group (CHO; n = 15). The control group was fed (100 g/day) of normal (NOR) diet for a period of 15 weeks. The HFD group was fed normal diet supplemented with 1.0% cholesterol plus 1.0% olive oil (100 g/day) for the same period of time.

**Results:**

We found that the TC, LDLC, and TG (mg/dl) were significantly (p < 0.001) increased in HFD rabbits compared with control rabbits with percentage normalized changes of 1198%, 1591%, and 710%, respectively. The peptide-bound Hyp in the serum was significantly (P < 0.05) increased in HFD rabbits compared with control rabbits with percentage normalized change of 517% while it significantly (P < 0.01) decreased in aortic tissues of HFD rabbits compared with control rabbits with percentage normalized change of 65%. The protein-bound Hyp in the serum was significantly (P < 0.01) increased in HFD rabbits compared with control rabbits with percentage normalized change of 100%; the protein-bound Hyp in the aortic tissues of control rabbits was 235.30 ± 55.14 (Mean ± SD) while it was not detectable (ND) in HFD rabbits. Total serum Hyp showed no significant (P < 0.05) change in HFD rabbits compared with control rabbits while it was significantly (P < 0.05) decreased in aortic tissues of HFD rabbits compared with control rabbits with percentage normalized change of 73%. The total collagen was significantly (p < 0.01) decreased in aortic tissues of HFD rabbits compared with control rabbits with percentage normalized change of 73% which was supported by histological study.

**Conclusions:**

These results suggest that percentage decrease in various Hyp fractions in aortic tissue of HFD rabbits are closely related to percentage decrease of collagen content in aortic tissues of HFD rabbits. These results also suggest that it may be possible to use the changes in various Hyp fractions in aortic tissues of rabbits as an important risk factor during the progression of atherosclerosis.

## Background

Gregory, 1999 [1] has postulated that serum hypercholesterolemia accelerates atherogenesis by augmenting cholesterol accumulation in the arterial intima. Hypercholesterolemia has been associated with an increased risk of coronary heart disease [2]. High TC and LDLC have been correlated with the increased risk of atherosclerosis [3,4]. Collagen represents the chief structural protein accounting for approximately 30% of all vertebrate protein. In majority of the tissues the most important function of collagen is a mechanical one - to withstand the mechanical parameters. The Hyp is a post translactional product of proline hydroxylation catalyzed by the enzyme prolylhydroxylase (EC 1.14.11.2) [5]. The occurrence of this amino acid is thought to be confined exclusively to collagen, where it is present in the Y position of the Gly-X-Y repeating tripeptide [6]. Consequently, the presence of Hyp in serum or tissues can be used as a measure of collagen or collagen degradation products [7]. Previous studies have reported that HgCl2 treatment to rats damages the collagen which is reflected by increased levels of Hyp in serum and an increased excretion of Hyp in urine [8,9]. Abdelhalim et al., 1994 [10] have reported that HFD has a general tendency to induce softening of the arterial wall due to denaturation of collagen and elastin which exist in the media of the arterial wall and is known to play a dominant role in governing mechanical properties of blood vessels. In the present study an attempt was made to clarify the changes in various Hyp fractions in serum and aortic tissue of HFD rabbits, and to clarify the changes in various Hyp fractions with the collagen content in aortic tissues of HFD rabbits and during the progression of atherosclerosis

## Materials and methods

### Aortic rabbit tissue samples

The atherosclerotic model used in this study was the New Zealand white rabbit (male, 12 weeks old), obtained from the Laboratory Animal Center (College of Pharmacy, King Saud University). Twenty five rabbits were individually caged, and were divided into control group and HFD group. The control group (NOR; n = 10) was fed on 100 g/day of NOR diet (Purina Certified Rabbit Chow # 5321; Research Diet Inc., New Jersey, USA) for a period of 15 weeks. Chemical composition of the laboratory NOR rabbit diet (Purina Certified Rabbit Chow # 5321). The HFD group (n = 15) was fed on NOR Purina Certified Rabbit Chow # 5321 supplemented with 1.0% cholesterol plus 1.0% olive oil (100 g/day) for the same period of time. The animals were sacrificed by intravenous injection of Hypnorm (0.3 ml/kg) into the auricular vein in accordance with the guidelines approved by King Saud University Local Animal Care and Use Committee. The thoracic aorta tissues of control and HFD rabbits were removed with great care so as to avoid any damage for aortic tissues, and were placed in 10% buffered neutral formalin. The thoracic aortic tissues were stored in a refrigerator at a temperature of 4°C for a period less than 48 hrs until the staining was performed. Other specimens of thoracic aortic tissues were preserved in liquid nitrogen to determine Hyp concentration.

### Collection of blood and preparation of serum

Blood samples of 2 ml were obtained from the rabbits via venepuncture of an antecubital vein. Blood was collected into two polypropylene tubes viz., one for serum and the other for plasma. The blood for plasma was collected in heparin. Serum was prepared by allowing the blood to clot at 37°C and centrifuge at 3000 rpm for ten minutes.

### Staining thoracic aortic tissue specimens

According to the routine procedures, thoracic aortic tissue specimens were stained by Victoria blue staining to examine fatty streaks, fibrous plaques, and any degenerative changes in collagen and elastin of the aortic tissues.

### Determination of total cholesterol and low-density lipoprotein cholesterol

Serum TC, LDLC, TG concentrations (mg/dl) were analyzed and determined by a clinical laboratory centre according to the previously reported method [11,12].

### Preparation of the sample for hydroxyproline estimation

Dissected aortic tissue specimens were homogenized in normal saline (0.8 percent g ml^-1^) using a stainless steel Omni-Mixer homogenizer (Omni International, Inc, Gainesville, VA, USA). The homogenate was used for determination of Hyp concentrations. Further details about sample collection have been previously reported by [13]. Total collagen content (mg/gm fresh tissue) was calculated from Hyp concentration assuming that Hyp constitutes 12.5% of collagen [14].

### Extraction of hydroxyproline fractions

Free and protein-bound Hyp were extracted by the method of Varghese et al., 1981 [15] with a slight modification as described by Siddiqi et al., 2000 [13] in which 0.5 ml of the homogenate was treated with 3 × 5 ml of rectified absolute alcohol and centrifuged at 3000 rpm for 10 min. The supernatant was pooled and kept at 40°C till the evaporation of ethanol. The residue was dissolved in 0.5 ml of distilled water, and 50 μl of the extract was used for estimation of free Hyp. The peptide-bound Hyp was determined after alkaline hydrolysis of the ethanol extractable fraction. The pellets of all the samples were dissolved in an aliquot of distilled water, and 50 μl of the extract was used for determination of protein-bound Hyp. The precipitate obtained upon ethanol treatment of the plasma was subjected to alkali hydrolysis to determine protein-bound Hyp. The total Hyp concentration (mg/g fresh tissue) was measured by the modified alkaline hydrolysis method of Reddy and Enwemeka, 1981 [7]. Briefly, 50 μl of homogenate sample was added to Na OH (2N final concentration), and the mixture was hydrolyzed by heating in boiling water bath for about 3 - 4 h. Approximately 900 μl of 56 mM chloramines T reagent was added to the hydrolyzed sample and oxidation was allowed to proceed at the room temperature for 25 min. Then 1.0 ml of 1 M Ehrlich's reagent (P- dimethylaminobenzaldehyde) was added to the oxidized sample and the chromophore was developed by incubating the samples at 65°C for 20 min. The absorbance was estimated at wavelength of 550 nm. The Hyp concentration in the samples was calculated from the standard curve of Hyp. More details about optimization, linearity, specificity, precision, and reproducibility have been previously reported [13].

### Statistical analysis

Statistical analysis was performed by means of InStat^® ^package for personal computers (GraphPad™ Software, Inc., San Diego, USA). Values were considered significant if P < 0.05. Each sample was run in duplicate. The Hyp concentration and collagen content were expressed as mean ± SD (μg or mg/g wet weight tissue for control rabbits: n = 10 animals; for HFD rabbits n = 15 animals). The Hyp concentration and collagen content in various aortic tissues of control and HFD rabbits were compared using one-way ANOVA analysis followed by Turkey's test for multiple comparisons. Bartlett's test was used for homogeneity of variances. Spearman correlation analysis was used to examine the association between variables.

## Results

**Figure 1:**
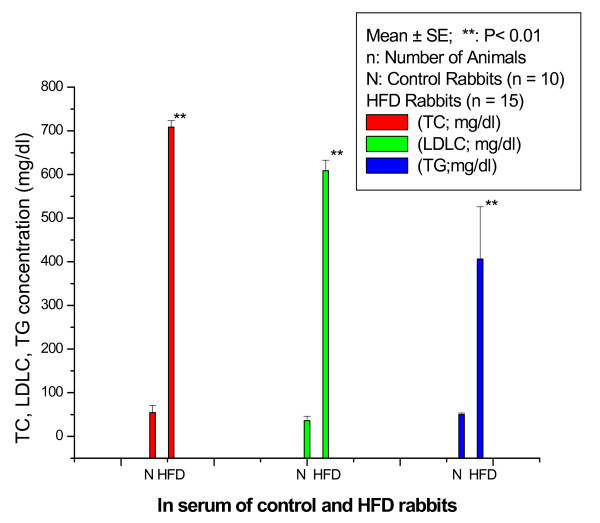
**TC, LDLC, and TG concentration (mg/dl) in the serum of control and HFD rabbits**.

Figure 1 shows TC, LDLC, and TG concentrations (mg/dl) in serum of control and HFD rabbits. The TC (mg/dl) significantly (p < 0.001) increased in HFD rabbits compared with control rabbits with percentage normalized change of 1198%. The LDLC (mg/dl) significantly (P < 0. 001) increased in HFD rabbits compared with control rabbits with percentage normalized change of 1591%. The TG (mg/dl) significantly increased in HFD rabbits compared with control rabbits with percentage normalized change of 710%.

Table 1 shows the concentration of various Hyp fractions in the serum of control and HFD rabbits. There was no significant (P < 0.05) decrease in free serum Hyp (μg/ml) in HFD rabbits compared with control rabbits. On the other hand, HFD rabbits showed a significant increase in serum peptide-bound (mg/ml) and protein-bound Hyp (mg/ml) with percentage normalized change of 517% (P < 0.05) and 100% (P < 0.01) respectively compared with control rabbits. However, total serum Hyp (mg/ml) in HFD rabbits showed no significant (P < 0.05) change when compared to control rabbits.

**Table 1:** Changes in various hydroxyproline fractions in the serum of control and HFD rabbits

Hydroxyproline fractions in the serum	Control rabbits (n = 10)	HFD rabbits (n = 15)
Free Hydroxyproline (μg/ml)	26.27 ± 5.66	16.19 ± 1.92^ns^

Peptide-bound Hydroxyproline (mg/ml)	27.16 ± 2.89	167.92 ± 72.30*

Protein-bound Hydroxyproline (mg/ml)	ND	95.57 ± 16.33**

Total Hydroxyproline (mg/ml)	156.33 ± 22.40	279.69 ± 68.67^ns^

*P < 0.05 as compared with control rabbits; **P < 0.01 as compared with control rabbits (Tukey's multiple comparison test); n: number of animals; data expressed as Mean ± SD; ND: not detectable; ns: not significant as compared with control rabbits; HFD rabbits: fed on HFD for 15 weeks).

Table 2 shows the concentration of various Hyp fractions in the aortic tissues of control and HFD rabbits. There was no significant (P < 0.05) decrease in free Hyp (μg/g fresh tissue) in the aortic tissues of HFD rabbits compared with control rabbits. The peptide-bound Hyp (mg/g fresh tissue) was significantly (P < 0.01) decreased in aortic tissues of HFD rabbits compared with control rabbits with percentage normalized change of 65%. In aortic tissues of control rabbits, the protein-bound Hyp (μg/g fresh tissue) was 235.3 ± 55.14 while it was not detectable in aortic tissues of HFD rabbits. The total Hyp (mg/g fresh tissue) was significantly (P < 0.05) decreased in aortic tissues of HFD rabbits compared with control rabbits with percentage normalized change of 73%.

**Table 2:** Concentration of various Hyp fractions in aortic tissues of control and HFD rabbits

Hydroxyproline fractions in the aortic tissues	Control rabbits (n = 10)	HFD rabbits (n = 15)
Free Hydroxyproline (μg/g fresh tissue)	147.30 ± 34.22	136.50 ± 32.15^ns^

Peptide-bound Hydroxyproline (mg/g fresh tissue)	5.24 ± 1.43	1.84 ± 0.62**

Protein-bound Hydroxyproline (μg/g fresh tissue)	235.30 ± 55.14	ND

Total Hydroxyproline (mg/g fresh tissue)	7.32 ± 3.46	1.96 ± 0.59*

*P < 0.05 as compared with control rabbits; **P < 0.01 as compared with control rabbits (Tukey's multiple comparison test); n: number of animals; data expressed as Mean ± SD; ND: not detectable; ns: not significant as compared with control rabbits; HFD rabbits: fed on HFD for 15 weeks).

**Figure 2:**
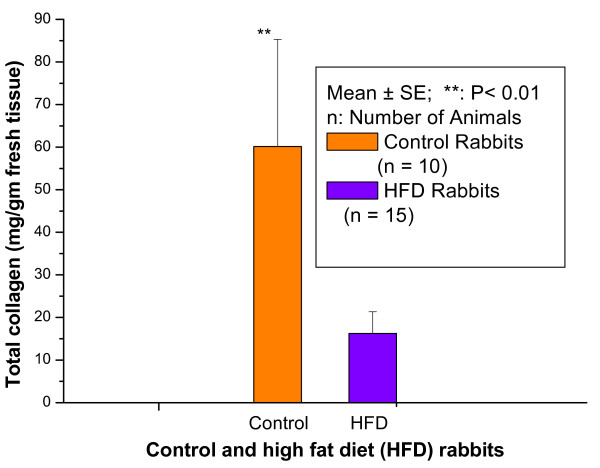
the total collagen (mg/gm fresh tissue) in aortic tissues of control and HFD rabbits

Figure 2 shows the total collagen (mg/gm) in aortic tissues of control and HFD rabbits. The total collagen was significantly (p < 0.01) decreased in HFD rabbits compared with control rabbits with percentage normalized change of 73%.

**Figure 3:**
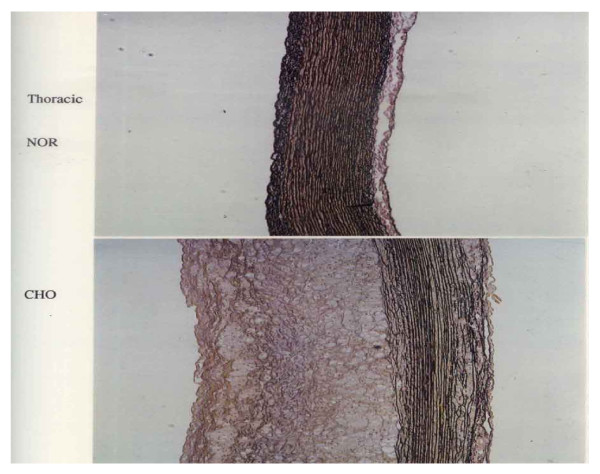
Photomicrograph of the Victoria blue stained thoracic aortic tissue obtained from a normal-fed rabbit (NOR) and a HFD (CHO)

Figure 3 represents photomicrograph of the Victoria blue stained thoracic aortic tissue obtained from a normal-fed rabbit (NOR) and a HFD rabbit (CHO). The upper panel (NOR) shows a normal thoracic aortic tissue morphology while the lower panel (CHO) shows marked intimal thickening, smooth muscle proliferation, connective tissue formation together with focal loss of normal medial architecture, and tunica media underlying plaques showed a marked disruption with loss of collagen and elastin fibers. The elastin and collagen fibers were found less condensed and fragmented near the innermost and outermost boundary of the media and within the central portion of the intima.

## Discussion

In the present study, the rabbits were fed a HFD diet containing 1% cholesterol plus 1% olive oil for a feeding period of 15 weeks. The accompanying changes in TC, LDLC, and TG concentration in serum of control and HFD rabbits were estimated. The various Hyp fractions in serum and aortic tissues of control and HFD rabbits and collagen content in aortic tissues of control and HFD rabbits were studied. Thoracic aortic tissue specimens were stained by Victoria blue to examine fibrous plaques and any degenerative changes in collagen and elastin of the aortic tissues of HFD rabbits. This study suggests that HFD has a general tendency to induce increase in TC, LDLC, and TG concentration which may be deposited in the atheromatous lesions, and may be incorporated into atherosclerotic plaques. Moreover, as another possible cause, it is suggested that HFD diet may accelerate atherogenesis through disturbing the mechanical fragility of atherosclerotic plaques making them vulnerable to rupture and thrombosis. It has been reported that when LDLC is oxidized by macrophages in lesions, it becomes toxic to the endothelium, and thereby could injure endothelial cells. Thus, the effects of HFD diet are not only confined to deposition of lipids in atheromatous lesions, but it may also produce primary endothelial cell injury [10]. In the present study, intima of the aortic tissues of HFD rabbits demonstrated a marked increase in thickness and smooth muscle cell proliferation. In addition, lipid laden cells were observed near the basement of the lesion, and the tunica media underlying plaques showed a marked disruption with a focal loss of collagen, elastin, and smooth muscle cells. The focal loss of collagen and elastin may induce softening of the arterial wall which is known to play a dominant role in governing mechanical properties of the blood vessels. It has been reported that feeding cholesterol to rabbits for a feeding period of 12 weeks induced softening of the arterial wall due to denaturation of collagen and elastin which exist in the media of the arterial wall [10]. Abdelhalim et al., 2010 [16] have reported that when specimens from the aorta of NOR and HFD rabbits fed on HFD for a period of feeding of 12 weeks were stained with Sudan, the aortic tissue specimens of control rabbits were completely free of fatty streaks and fibrous plaques, and were characterized by a barely visible intima. On the contrary, the aortic tissue specimens from HFD rabbits exhibited lesions which comprised of fatty streaks and fibrous plaques.

These results were further confirmed by biochemical analysis which showed that feeding rabbits with a HFD caused a significant decrease of Hyp fractions and collagen content in aortic tissues of HFD rabbits compared with control rabbits. In the present study there was no significant change in free Hyp in serum and aortic tissues of HFD rabbits compared with control rabbits. This shows that the processes which contribute to free Hyp pool viz., mature collagen, newly synthesized collagen dietary collagen etc. were not affected by HFD diet. There was a significant decrease in peptide-bound Hyp and protein-bound Hyp was not detected in aortic tissues of HFD rabbits compared with control rabbits.

Type I and III collagen plays an important role in arterial physiology by preventing arterial expansion beyond physiologic limits. Smooth muscle cells in the atherosclerotic arteries synthesize new extracellular matrix components, including collagen [17,18]. In atherosclerotic arteries, collagen is crucial for plaque stability and its removal from the plaque's fibrous cap area may result in plaque rupture [19]. In addition to providing the extracellular matrix stability, the extracellular collagen network functions as a framework for the migration of smooth muscle cells into the intima where they proliferate and synthesize new extracellular matrix components.

The aortic tissue is among the most abundant tissue sources of collagen XVIII [20] and also contains Type I and Type III collagen. Karen et al., 2004 [21] have hypothesized that collagen XVIII is degraded during atherosclerosis and that loss of this vessel wall proteoglycan promotes the proliferation of vasa vasorum into the intima of atheromas. There studies provide genetic evidence that loss of collagen XVIII promotes atherosclerosis. Loss of collagen XVIII increases plaque angiogenesis and vascular permeability to lipids by distinct mechanisms that develop at different gene doses. Studies of Karen et al., 2004 [21] also demonstrate that the function of collagen XVIII in basement membranes is to maintain vascular permeability. Since collagen plays important roles in plaque stability and cell migration properties, a comprehensive understanding of collagen expression and organization during the progression of atherosclerosis is essential.

## Conclusions

Twenty five rabbits were individually caged, and divided into control group and HFD group. The control group was fed of normal (NOR) diet for a period of 15 weeks. The HFD group was fed normal diet supplemented with 1.0% cholesterol plus 1.0% olive oil for the same period of time. The TC, LDLC, and TG were determined in the serum of control and HFD rabbits. The various Hyp fractions were determined in the serum of control and HFD rabbits, and the various Hyp fractions and collagen content were determined in aortic tissues of control and HFD rabbits.

We found that the TC, LDLC, and TG (mg/dl) were significantly (p < 0.001) increased in HFD rabbits compared with control rabbits with percentage normalized changes of 1198%, 1591%, and 710%, respectively. The peptide-bound Hyp in the serum was significantly (P < 0.05) increased in HFD rabbits compared with control rabbits with percentage normalized change of 517% while it significantly (P < 0.01) decreased in aortic tissues of HFD rabbits compared with control rabbits with percentage normalized change of 65%. The protein-bound Hyp in the serum was significantly (P < 0.01) increased in HFD rabbits compared with control rabbits with percentage normalized change of 100%; the protein-bound Hyp in the aortic tissues of control rabbits was 235.30 ± 55.14 (Mean ± SD) while it was not detectable (ND) in HFD rabbits. Total serum Hyp showed no significant (P < 0.05) change in HFD rabbits compared with control rabbits while it was significantly (P < 0.05) decreased in aortic tissues of HFD rabbits compared with control rabbits with percentage normalized change of 73%. The total collagen was significantly (p < 0.01) decreased in aortic tissues of HFD rabbits compared with control rabbits with percentage normalized change of 73% which was supported by histological study. These results suggest that percentage decrease in various Hyp fractions in aortic tissue of HFD rabbits are closely related to percentage decrease of collagen content in aortic tissues of HFD rabbits. These results also suggest that it may be possible to use the changes in various Hyp fractions in aortic tissues of rabbits as an important risk factor during the progression of atherosclerosis.

## Abbreviations

TC: total cholesterol; LDLC: low-density lipoprotein cholesterol; TG: triglycerides; Hyp: hydroxyproline; NOR: normal; HFD (CHO): high fat diet; ND: not detectible; ns: not significant.

## Competing interests

The authors declare that they have no competing interests.

## Authors' contributions

MAKA, NJS, ASA and MSAA have participated in all experiments, data interpretation and analysis and drafting the manuscript. The atherosclerotic model used in this study was obtained from the Laboratory Animal Center (College of Pharmacy, King Saud University). The control and HFD was prepared by Research Diet Inc., New Jersey; USA. The authors have conceived the study and its design, supervised all technical activities, and written the final version of the manuscript. All authors have read and approved the final manuscript.
